# Salivary Diagnostics—Point-of-Care diagnostics of MMP-8 in dentistry and medicine

**DOI:** 10.3390/diagnostics7010007

**Published:** 2017-01-20

**Authors:** Nilminie Rathnayake, Dirk-Rolf Gieselmann, Anna Maria Heikkinen, Taina Tervahartiala, Timo Sorsa

**Affiliations:** 1 Division of Periodontology, Department of Dental Medicine, Karolinska Institutet, 17177 Stockholm, Sweden; 2 Division of Periodontology, Dentognostics GmbH, 07745 Jena, Germany; gieselmann4@aol.com; 3 Department of Oral and Maxillofacial Diseases, University of Helsinki, Helsinki University Central Hospital, 00014 Helsinki, Finland; anna.m.heikkinen@helsinki.fi (A.M.H.); taina.tervahartiala@helsinki.fi (T.T.); timo.sorsa@helsinki.fi (T.S.)

**Keywords:** diagnostic test, point-of-care, periodontitis, peri-implantitis, systemic diseases

## Abstract

Human saliva is an easily accessible biological fluid and contains a variety of disease-related biomarkers, which makes it a potential diagnostic medium. The clinical use of salivary/oral fluid biomarkers to identify oral and systemic conditions requires the development of non-invasive screening and diagnostic technologies, and is among the main goals of oral fluid researchers. The analysis of the disease-specific oral and systemic biomarkers in saliva and oral fluids (i.e., mouth-rinse, gingival crevicular fluid (GCF) and peri-implantitis sulcular fluid (PISF)) is demanding. Several factors influence their expression and release; these factors include the intracellular location, the molecular size and the flow characteristics of the biological fluid. The type of saliva/oral fluid utilized for the diagnostics affects the analysis. High sensitivity together with sophisticated methods and techniques are essential to get a useful outcome. We describe here a recently developed mouth-rinse that is practical, convenient and inexpensive, as well as PISF chair-side/point of care (PoC) lateral-flow active matrix metalloproteinase (aMMP-8) immunoassays to detect, predict and monitor the course and treatment of periodontitis and peri-implantitis.

## 1. Introduction

**Figure 1:**
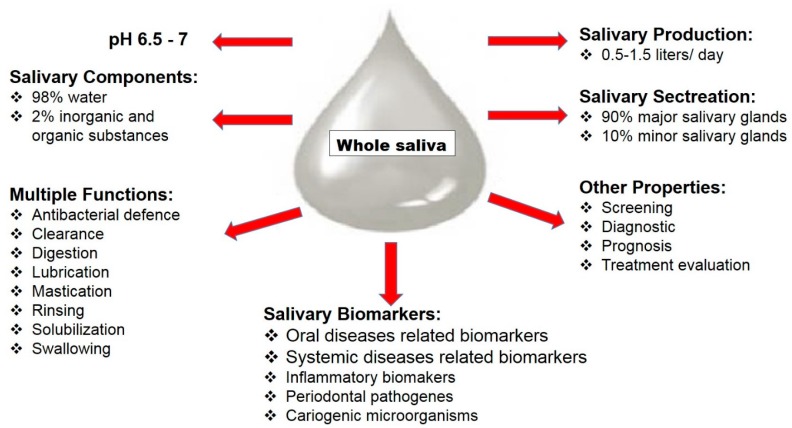
Components, properties and secretion of human whole saliva.

Human saliva is an easily accessible biological fluid and contains a variety of disease-related biomarkers, which makes it a potential diagnostic medium. Whole saliva is secreted from three pairs of major salivary glands—the parotid, submandibular and sublingual glands—and numerous minor salivary glands from non-glandular sources, such as gingival crevicular fluid (GCF) [1]. Salivary production per day is 0.5 to 1.5 L in normal conditions, and saliva’s components are 98% water and the 2% electrolytes, mucus, antibacterial compounds, and various enzymes. This unique oral fluid has multiple functions, such as rinsing, solubilization of food substances, food and bacterial clearance, lubrication of soft tissues, bolus formation, dilution of detritus, swallowing, speech and facilitation of mastication, all of which are related to its fluid characteristics and specific components. In addition, saliva components contribute to mucosal coating, digestion and antibacterial defence (Figure 1) [2]. Furthermore, inflammatory biomarkers associated with oral and common systemic diseases have been identified in saliva: interleukins-1β, -6 and -8 (IL-1β, -6 and -8), tumour necrosis factor-a (TNF-α), matrix metalloproteinases (MMP)-8 and -9, and tissue inhibitors of metalloproteinase (TIMP)-1 [3,4].

### 1.1. Saliva as a Diagnostic Medium

Utilization of inflammatory and disease-specific biomarkers in saliva could offer an attractive solution for screening or diagnosis of different conditions. Salivary composition essentially originates from blood, but in the salivary glands, active transport and secretion mechanisms may change the saliva composition as the organic components of glandular specific saliva are derived from protein synthesis and are stored within the acinar cells [5,6]. Based on this biological mechanism, saliva could be an alternative to plasma/serum analysis for screening, diagnostic and prognostic purposes as well as evaluation of treatment outcome. The biggest advantage of using saliva is that collection is non-invasive and a plausible method.

### 1.2. Salivary Biomarkers and Oral Diseases

Local inflammation is characterized by being present in an isolated area of the body; in this case it is the oral cavity and is called oral inflammation. Inflammatory mediators are released from different cells due to inflammatory conditions and the inflammatory biomarkers associated with oral diseases have been analyzed in saliva samples, like interleukins, tumour necrosis factors, lysozymes, matrix metalloproteinases, tissue inhibitors of metalloproteinases, myeloperoxidase and the total protein contain [3,5,7].

### 1.3. Salivary Biomarkers and Periodontal Disease

The term “periodontal disease” encompasses gingivitis, chronic periodontitis and aggressive periodontitis, and there are numerous subcategories as well: periodontitis as a manifestation of systemic disease, necrotizing periodontal disease, abscesses of the periodontium, periodontitis associated with endodontic lesions, and development of acquired deformities and conditions, according to the International Workshop for the Classification of Periodontal Disease [8]. There are two forms of gingivitis: plaque-induced gingivitis and non-plaque-induced gingivitis. The most common type is plaque-induced gingivitis with the presence of inflammation in the gum. Clinical features of plaque-induced gingivitis are redness, swelling, and bleeding. This condition is reversible, usually be treatment and adequate oral care maintenance [8].

Periodontitis is the sixth most common chronic inflammatory disease in the world [9]. It is a multifactorial condition and is associated with complex interactions between periodontal bacteria (*Porphromonas gingivalis*, *Tannerella forsythia* and *Treponema denticola*, together with *Aggregatibacter actinomycetemcomitans*), the host inflammatory response, and genetic, environmental and behavioral risk factors. The most common form of the disease is plaque-induced periodontitis, characterized by gingival inflammation, release of different pro-inflammatory cytokines, and destruction of periodontal tissues and alveolar bone. Pocket formation and swollen and bleeding gingiva are the clinical signs of periodontitis. Chronic periodontitis is a slowly progressing disease but it can include episodes of more rapid progress. The supporting collagen of the periodontium degenerates, resorbing alveolar bone, and the gingival epithelium migrates along the tooth surface, ultimately forming a periodontal lesion. Finally, the outcome of untreated periodontitis is tooth loss [10,11]. Diagnosis of periodontal disease is based upon clinical examination and radiographical assessments of periodontal tissues but tools for screening/diagnosis, evaluation of severity and prognosis of periodontal disease are presently insufficient. Based on this, disease-specific biomarkers in saliva as a complement to regular clinical and radiographical examinations are of interest, in particular for point-of-care (PoC) periodontitis tests.

Whole saliva contains local- and systemic-derivative biomarkers, which raises the possibility of using saliva as a diagnostic medium for periodontal disease. Inflammatory biomarkers, such as IL-1β, IL-6, and IL-8, MMP-8, TIMP-1 and TNF-α associated with oral diseases: dental caries, gingivitis and periodontitis have been detected in saliva [3,4,7,12].

In inflammatory disorders, matrix metalloproteinases (MMPs) are highly involved. In humans, twenty-three genetically distinct MMPs have been identified; they are calcium-dependent zinc containing endopeptidases that play an important role in tissue development and remodeling as well as in pathological processes [13]. MMPs are involved in the pathogenesis of a large number of different diseases and conditions as they have an anti-inflammatory and tissue destructive role and [14]. MMPs are produced in different forms, in latent, non-active pro-forms, and are activated extra- or intracellularly depending on the structure of the MMP molecules [15]. Tissue inhibitors of metalloproteinases (TIMPs) are the main inhibitors of MMPs that control the extracellular matrix component breakdown [16]. Among all MMPs, MMP-8 and -9 are associated with periodontal disease according to previous studies [17]. Neutrophil collagenase/MMP-8 released due to inflammatory conditions by neutrophils, endothelial and smooth muscle cells and macrophages [17]. Previous investigations showed that salivary MMP-8 levels are associated with progressive loss of attachment in periodontitis [17,18]. In addition, salivary levels of IL-1β and MMP-8 are significantly associated with severe periodontitis compared to healthy controls [7].

### 1.4. Salivary Biomarkers and Peri-Implantitis

Peri-implantitis is an inflammation in peri-implant tissues and loss of supporting alveolar bone. The overall prevalence of peri-implantitis is 14%–30% according to a systematic review [19], and several cross-sectional studies conducted in Sweden reported that moderate and severe forms of peri-implantitis occurred in subgroups of 15%–20% of implant-carrying subjects [20,21]. The inflammation in peri-implantitis lesions are more aggressive compared to periodontal lesions, so prevention of peri-implantitis is a high priority [22]. Mucositis is the precursor to peri-implantitis, and the progression is from healthy implant mucosa to mucositis and finally to peri-implantitis. Preventive care, treatment and management of mucositis prevent the transformation of mucositis to peri-implantitis [22]. Such assessments are difficult since they entail early detection and signs of loss of supporting tissues. So far, peri-implant bone loss and the progression pattern of the disease have been evaluated by radiographs in patients with severe forms of peri-implantitis.

Number of studies reveal that oral fluid biomarkers could be used to discover peri-implantitis [23,24]. Mouth rinsing could offer a low-cost and non-invasive method for collecting oral fluid, in particular for adjunctive point-of-care (PoC) peri-implantitis diagnostics [25]. In peri-implantitis lesions, MMP-8 is a major destructive collagenase [25] and results from previous studies showed that MMP-8 in oral fluids could have predictive value [26,27,28,29]. In addition, the progression of peri-implantitis has been repeatedly associated with pathologically excessive elevation of MMP-8 in oral fluids [30]. There are some studies that have measured MMP-8 in implant sulcus fluid, GCF and saliva [31,32] but there is a gap in knowledge based on the utility of a POC oral fluid MMP-8 test for chair-side diagnostics of peri-implantitis.

### 1.5. Salivary Biomarkers and Dental Caries

Dental caries is a multifactorial infectious disease and is highly prevalent around the world [33]. Cariogenic microorganisms in the oral biofilm is the main cause of dental caries. Saliva has multiple factors that protect the teeth not to get decayed [34]. The most common way of diagnosis dental caries is based to clinical- and radographical examinations as well as measurement of salivary flow rate. Saliva has different measurable biomarkers that could be used for diagnosis, prediction, prognosis, management and evaluating the outcome of therapeutic regimens. Dental caries-associated pathogens have been detected in saliva, such as *Streptococcus mutans*, *Streptococcus sobrinus* [35], *Lactobacilli* [36], *Streptococcus sanguinis*, *Streptococcus salivarius* [37], *Actinomyces spp* [38], *Veillonella* [39] and *Candida albicans* [40]. Salivary electrolytes biomarkers, namely calcium, fluoride, phosphate and bicarbonate, are considered to be highly important for protecting teeth from dental caries [41]. Additional human studies are needed to strengthen the statement of the anti-caries effect contributed by naturally occurring salivary electrolytes. Immunoglobulins (Ig) are the major group of proteins appearing in human saliva. The prominent immunogloblins in saliva are a subclass of IgA, followed by IgG and IgM subclasses [42]. In addition, there are a number of innate host defense proteins and peptides that could be used as salivary biomarkers for dental caries, such as agglutinins, amylase, antimicrobial peptides, lysozymes, lactoferrin, mucous glycoproteins, peroxidase and total protein level [43,44,45,46]. The salivary flow rate, pH in saliva and buffering capacity and salivary sugar clearance rate are also considerable salivary biomarkers for detection of dental caries lesions [47]. Inflammatory biomarkers associated with dental caries have been detected in saliva [48].

### 1.6. Salivary Biomarkers and Systemic Inflammation

Systemic inflammation has acute and chronic forms, and the biochemical processes release cytokines as “emergency signals” that bring in the body's immune cells and activate the innate immune system, hormones and nutrients to solve the problem. A number of specific molecular biomarkers for different conditions, such as cancer, diabetes and cardiovascular disease (CVD)/myocardial infarction (MI), are being identified [49,50,51].

### 1.7. Salivary Biomarkers and Cancer

In salivary diagnostics, to detect or monitor different types of cancer is a main focus of the research field. Oral squamous cell carcinoma (OSCC) is the most common malignant neoplasm of the oral cavity [52]. OSCC patients indicated that a specific marker of oxidative stress, malondialdehyde (MDA) in saliva, is a better diagnostic tool as compared to MDA in blood [53]. IL-8 levels in saliva were elevated in patients who had experienced tumour diseases [3]. Salazar et al. (2014) reported to detect head and neck squamous cell carcinoma (HNSCC) MicroRNAs (miRNAs) of saliva were used, and the results showed that miR-9, miR-134 and miR-191 were differentially expressed between saliva from HNSCC patients and healthy controls. The same research group suggested that these saliva-derived miRNAs may serve as novel biomarkers to reliably detect HNSCC [54]. A number of cytokines and chemokins involved in cancer progression are detectable in saliva-based testing of these biomarkers and are promising. They depend upon the methods/techniques for analysis, such as interferon-gamma (IFN-γ), TNF-α, IL-1β, transforming growth factor-beta-1 (TGF-β1), epidermal growth factor (EGF), IL-6 and -8, vascular endothelial growth factor (VEGF), interleukins-4 and -10, tumour necrosis factor (TNF) and endothelin [55]. Salivary gland carcinomas (SGCs) make up about 5% of all cancers of the head and neck, so there is a need to develop new molecular biomarkers for early and improved diagnosis of SGCs and further research in this area is required.

### 1.8. Salivary Biomarkers and Diabetes

One of the most common chronic diseases is diabetes, which occurs either when the pancreas does not produce enough insulin or when the body cannot effectively use the insulin it produces. Hyperglycaemia is an effect of uncontrolled diabetes and over time leads to serious damage to several organs in the body, mainly the blood vessels and nerves [56]. The prevalence of diabetes in adults over 18 years of age was 8.5% in 2014 worldwide and the prevalence has been increasing rapidly in middle- and low-income countries [57]. Diabetes is a major cause of blindness, kidney failure, myocardial infarction, stroke and lower limb amputation [57] and has an association with periodontal disease [58]. Few studies are available that detect salivary inflammatory biomarkers in patients with diabetes. In a child population, unstimulated saliva samples were analyzed and the salivary levels of CRP, insulin and leptin are remarkably higher in obese children compared to healthy, normal-weight children [59]. In a cross sectional study consisting of 451 patients Rathnayake et al. (2013) found elevated salivary levels of MMP-8 among diabetes patients [3]. In type I diabetes patients, salivary *N*-acetyl-β-d-hexosaminidase was found to be significant increased compared to healthy control subjects [60]. In a study performed by Border and co-author, a reported 52 different proteins were detected, and some highly diabetes-related salivary inflammatory biomarkers were observed in diabetes patients compared to controls [61].

### 1.9. Salivary Biomarkers and Myocardial Infarction

In 1956, a study on the use of cardiac biomarkers of myocardial infarction (MI) was published, [62] and since that time, highly sensitive methods for biomarker detection have been developed. Due to a myocardial injury caused by myocardial ischemia- and necrosis certain biomarkers are released, such as cardiac troponins I (TnI) and T (TnT), creatine kinase-MB, total creatine kinase, myoglobin, lactate dehydrogenase [63,64]. Cardiac TnI and TnT consider as the golden standard for diagnosis of acute MI (AMI) as they are tissue specific for the myocardium [65]. Few publications have revealed correlations between serum and salivary biomarkers related to cardiovascular disease [66,67]. Tn I levels reach their peak within 10–14 h following an AMI, and according to previous studies, Tn I levels could be detected in saliva within 24 h of onset of AMI [67]. Floriano et al. (2009) showed in their investigation that saliva-based nano-biochip tests together with an electrocardiogram could provide a prompt screening method for AMI patients in the prehospital stage, and the investigators in this study were also able to detect elevated salivary levels of creatine kinase-MB, myoglobin, TnI and T, C-reactive proteins (CRP), TNF-α, MMP-9 and myeloperoxidase in AMI patients [68]. Furthermore, a few other markers, like cystatin C, growth differentiation factor-15 and N-terminal prohormone of brain natriuretic peptide related to MI are detectable in saliva [69]. In a Finnish study, patients with acute ischemic stroke had their systemic and local inflammatory markers analyzed in saliva. The results of this study showed controls had enhanced levels of salivary MMP-8, myeloperoxidase (MPO) and IL-1β compared to the patients, since the control group was suffering from ongoing periodontal disease and the patients more often had evidence of end-stage periodontitis with edentulism and missing teeth. In addition, the patients in this study had higher levels of serum MMP-8 and MPO [70]. Additional longitudinal studies are needed to check the potential for detecting salivary biomarkers associated with myocardial infarction and ischemic stroke.

## 2. Development of Point of Care/Chairside Test for Oral (Periodontal Disease and Peri-Implantitis) Conditions

Developing a chairside/PoC-based to disease-specific biomarkers is of great interest as it would make life easier for clinicians and researchers in odontological science as well as for patients. Disease-specific biomarkers increase the specificity and sensitivity when obtaining diagnostic and prognostic information.

Specificity and sensitivity needed to be considered when it comes to usefulness of salivary diagnostic/screening tests in clinics. The sensitivity of a test describes its capacity to properly identify patients with the disease. It is very important to have highly sensitivity tests to use to identify different outcomes, e.g., a clinical test with 75% sensitivity identifies 75% of patients with the disease (true positives) but 25% with the disease will be undetected (false negatives). Specificity of a test describes the capacity of the clinical test to properly identify patients without the disease, e.g., a clinical test with 75% specificity appropriately shows 75% of patients without the disease as test negative (true negatives) but 25% of patients without the disease are falsely identified as positive (false positives). A clinical test with high specificity and sensitivity is required to obtain acceptable outcomes.

PerioSafe [26] and ImplantSafe are two ideal PoC tests that are rapid, sensitive, accurate, reproducible and inexpensive. These are two different chair-side mouth rinse and PISF tests based on an active MMP-8 (aMMP-8) immunoassay for measuring the inflammatory burden of periodontitis and peri-implantitis.

### 2.1. PerioSafe

PerioSafe, the lateral flow chromatography aMMP-8 oral fluid PoC–immunotest, identifies and screens chronic and initial periodontitis sites and patients, differentiates active sites and patients, predicts disease progression, and can be utilized to monitoring the treatment and medication as well as during maintenance [27,28,29,71,72]. Additionally, it identifies genetically predisposed adolescents [73]. A positive aMMP-8 test is based on a cutoff of 25 ng aMMMp-8 per milliliter of filtrate derived from 5 mL mouth rinse. After comparing values from 130 patients with chronic periodontitis at six different cutoffs (20, 25, 30, 35, 40 and 50 ng/mL) by ELISA, the cutoff of 25 ng/mL was chosen based on ĸ values [71]. One line on the test device indicates that the test has successfully analyzed the drop of mouth rinse, and the result is negative. The result is positive if two lines are observed, indicating elevated risk of periodontitis [71].

The sensitivity and specificity of the PerioSafe aMMP-8 test have been demonstrated to be 76.5% and 96.7% for >2 sites and deepened pockets, respectively [71]. The test has been independently and internationally successful validated in Africa, Europe and USA, and in all studies it found to excellently differentiate periodontal health and disease [71,73,74,75,76].

**Figure 2:**
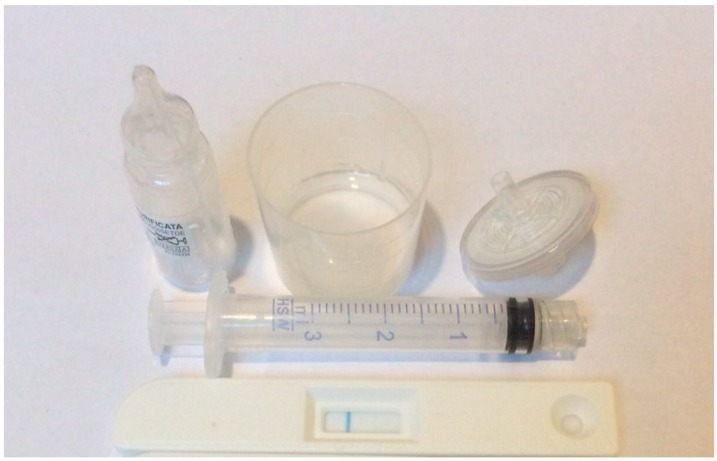
Salivary test kit used to detect the presence of aMMP-8 in periodontitis patients.

**Figure 3:**
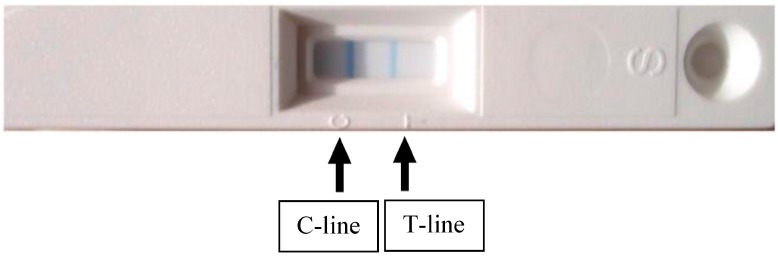
Two lines (Control line; C-line and Test line; T-line) due to elevated saliva aMMP-8 indicate elevated risk for periodontitis.

The aMMP-8 chairside test (Figure 2 and Figure 3) is performed on participants to identify individuals with elevated saliva MMP-8 in periodontitis patients. One line on the test device indicates that the test has successfully analyzed the drop of saliva and the result is negative. The result is positive if two lines are observed (Figure 3, indicating elevated risk for periodontitis; low-risk (light line) and high-risk (dark line). A test with 100% sensitivity recognize all patients with the disease; a test with 100% specificity rules out the disease in all healthy patients.

### 2.2. ImplantSafe

The ImplantSafe aMMP-8 (also a rapid lateral flow chromatography immunotest), is a modern, in-vitro diagnostic dip-stick test for use in dental implantology. This PoC test is a rapid test for routine implant checkups as a part of a regular implant maintenance program. The test can provide valuable information for preventive care through early detection of a risk of hidden inflammation with consequent tissue and alveolar bone break-down. Early detection provides the opportunity for timely treatment to arrest the development of mucositis or peri-implantitis. The sample is collected atraumatically directly at the implant, using a sterile peri-implant sulcus fluid (PISF) collection strip provided with the test. The results show elevated collagenase values (aMMP-8) in the peri-implant sulcus fluid. This natural enzyme breaks down tissue and is secreted early on the event of periodontal/peri-implant inflammations.

The test reveals elevated concentrations of aMMP-8 in PISF samples. This rapid test is based on a lateral flow sandwich immunoassay (DIPSTICK test) using the highly specific monoclonal antibodies MoAB 8706 and MoAB 8708, conjugated to latex particles.

A negative test result (only the control line appears) together with negative clinical diagnosis from the dentist suggests no or only minor risk of peri-implant inflammation with risk of break down. That why a negative test result during recall/maintenance can mean that the prophylactic measures like implant cleaning and maintenance visits were successfully carried out and within the right time window. It can also mean that the measures taken to treat a case of mucositis/peri-implantitis were successful.

**Figure 4:**
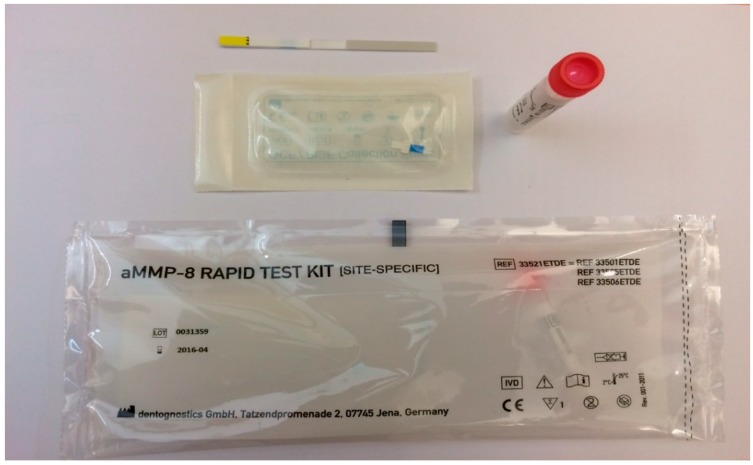
ImplantSafe rapid test kit to detect peri-implantitis.

**Figure 5:**
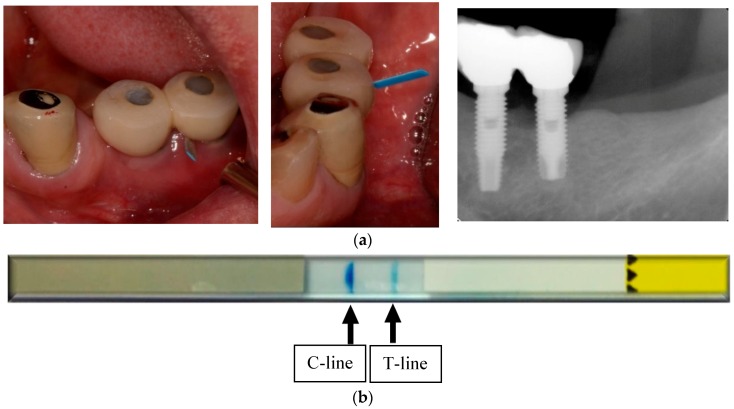
(**a**) Demonstrate the clinical and radiographic features of peri-implantitis and use of ImplantSafe; (**b**) Two clear lines indicate elevated risk for peri-implantitis. A positive test result: both control (C) line and test (T) line are visible, reveals elevated collagenolytic activity.

A positive test result (test line and control line are both visible) reveals elevated collagenolytic activity. This suggests an elevated risk of peri-implant tissue destruction and alveolar bone loss due, for example, to inflammatory processes. The test result should be evaluated by the treating dentist in light of the situation of the individual patient. Other parameters must be taken into consideration, such as age of the implant, bleeding on probing (BOP), probing pocket depth (PPD) and radiological evaluation of bone loss. Evidence of bruxism or abnormalities of occlusion should be assesses as well as general medical factors, like pregnancy or systemic illness (diabetes mellitus, rheumatic diseases, cardiovascular diseases, etc.). Implants that test positively should be monitored very closely, e.g., every three months. Elevated aMMP-8 values suggest an existing progression risk. In addition, even a weak test line indicates risk (Figure 4 and Figure 5a,b).

## 3. Conclusions

Certain biomarkers found in saliva are of high sensitivity and specificity, particularly in oral diseases, such as periodontal disease, dental caries and oral cancer, but identifying disease-specific molecular biomarkers in whole saliva is challenging, since advanced methods are required. Intracellular location, the size of the proteins, and the characteristics of the local biological fluid flow are factors that have an influence on the expression and release of biomarkers. In addition, the type of saliva used for diagnostic purposes to detect systemic conditions has an impact. In this regard, unstimulated saliva reveals more information than stimulated saliva since unstimulated saliva contains higher concentrations of screening/diagnostic biomarkers. Studies on the relationship between salivary biomarkers and oral- and systemic diseases have several methodological limitations that make it difficult to draw conclusions, as most of the studies are cross-sectional with a small number of subjects. As a results, this limit the statistical power and the possibility of establishing any causal relationship between analyzed biomarkers in saliva and certain conditions.

The current knowledge regarding the relationship between salivary biomarkers and disease diagnostics is limited, so the clinical utilization of oral fluid biomarkers to identify oral and systemic conditions calls for the development of non-invasive screening and diagnostic procedures. This is among the key goals of salivary/mouth-rinse/oral fluid researchers. Eventually this research field shall be designated “dental or oral clinical chemistry.” The aMMP-8 lateral-flow PoC immunotests (PerioSafe and ImplantSafe kits), currently commercially available, are good, inexpensive and practical examples of such developments in this field.
